# Intimate partner violence and common mental disorders among women in Mozambique: Evidence from the 2022-2023 Mozambique Demographic and Health Survey

**DOI:** 10.1371/journal.pmen.0000660

**Published:** 2026-10-13

**Authors:** Bernard Afriyie Owusu, Benedict Weobong

**Affiliations:** 1 Department of Social and Behavioural Sciences, School of Public Health, University of Ghana, Accra, Ghana; 2 African Mental Health Research Initiative (AMARI-II), West African Hub, University of Ghana, Accra, Ghana; Universidad Técnica de Manabí: Universidad Tecnica de Manabi, ECUADOR

## Abstract

Intimate partner violence (IPV) is a major public health concern and a violation of fundamental human right which disproportionately affects women and profoundly impacts mental health. This study used the 2023 Mozambique Demographic and Health Survey data to examine the association between IPV and common mental disorders (CMDs) among 2,790 reproductive-aged women in union. A composite outcome variable (CMDs), from either probable depression or anxiety, was computed using standardised thresholds. The main exposure was IPV, a composite focused on three forms: physical, emotional, and sexual, stratified by never, past, or recent exposures. A weighted binary logistic regression was performed in STATA 18/MP. Adjusted odds ratios (aOR) and their corresponding 95% confidence intervals were reported. The results show that the prevalence of probable depression and anxiety was 8.6% (95% CI: 7.1–10.5) and 10.7% (95% CI: 8.7–12.9) respectively. Overall, the prevalence of CMDs was 13.5% (95% CI: 11.4–15.9), comorbid symptoms was 5.8% (95% CI: 4.5 – 7.5). The prevalence of emotional, physical, and sexual IPV was 16.1%, 12.0%, and 4.0% respectively. Women who experienced any recent IPV (aOR = 2.05, 95% CI: 1.36 – 3.09), or past IPV (aOR = 1.40 CI: 0.80 – 2.44) had higher odds of CMDs compared to those who did not experience IPV. After adjusting for confounders, emotional IPV (aOR = 2.34, 95% CI: 1.6–3.4) remained independently associated with CMDs; physical (aOR=1.21, 95% CI: 0.73 – 2.03) and sexual IPV (1.67, 95% CI: 0.83 – 3.35) were not. Higher household wealth was protective against CMDs (aOR = 0.34, 95% CI: 0.13 – 0.88), and joint household decision-making was associated with higher odds of CMDs (aOR = 1.84, 95% CI: 1.08 – 3.15). The results demonstrate an interaction between marital status and IPV on CMDs, providing partial empirical support for Intersectionality theory. These findings underscore the need for regular mental health support and targeted IPV campaigns, particularly against emotional abuse among partnered women in Mozambique.

## Introduction

Intimate partner violence (IPV) is a major public health issue and a violation of fundamental human right which profoundly impacts mental health. Studies demonstrate a strong relationship between various forms of IPV, notably physical, sexual, and emotional, and common mental disorders (CMDs) including depression and anxiety [1–3]. IPV, like CMDs, disproportionally affect women globally [3], and creates a vicious cycle which increases women’s vulnerability to further violence through impaired coping or perpetrator control [4,5].

Across the globe, nearly 1 in 3 women experience physical and/or sexual IPV [6]. The prevalence of IPV range from 20% in the Western Pacific, 22% in high-income countries, and 33% in the WHO African region [6]. Regional variations show higher rates in the WHO African region including Mozambique where about 33% of women have experienced lifetime IPV [7]. Common Mental Disorders including probable depression and anxiety affect about 12% and 30% of ever-married women in Mozambique respectively [2], with emotional IPV being the most prevalent at 22.4%, followed by physical IPV at 21.3% [7].

Reproductive-aged women (15–49 years) face unique vulnerabilities to IPV due to multiple biological, economic, and socio-cultural factors including power imbalances and patriarchy [8]. In many low and middle-income countries, factors such as lower household wealth, rural residency, limited decision-making power, family violence, unemployment, educational gaps, and harmful masculine behaviours correlates with higher odds of CMDs [1,8,9]. Theoretical narratives suggest that these factors provide different effects on the mental health burden of IPV, highlighting the importance of intersectional perspectives in understanding women’s vulnerability to IPV [10]. Women who have experienced IPV risk repeat victimisation, with 15–70 percent of them re-abused by the same partners [11]. IPV and CMDs often moderate trauma pathways and suicidality [12], with survivors facing higher risks of unplanned pregnancies, induced abortion, gynaecological symptoms, STIs including HIV, menopausal symptoms, cardiovascular and endocrine disorders, and multiple neurological symptoms including migraine and loss of consciousness [13–17].

This study draws on the Ecological Model of Violence (EMV) and Intersectionality theories to conceptualise and understand the drivers of IPV among partnered women in Mozambique. The EMV, based partly on Bronfenbrenner’s ecological systems theory is a theoretical framework used by Heise (1998) to explain how violence results from factors operating at multiple individual, relational, community, and societal levels [18]. Also, although, traditional theories on IPV focuses on patriarchy as the root cause of violence against women, Intersectionality as coined by Crenshaw (1989) challenges this narrative. Intersectionality posits that a woman’s experience of IPV is not shaped solely by gender or patriarchy, but by the overlapping influences of multiple identities including class, culture, and age. Among the key principles of intersectionality are power interaction, context, and multidimensional inequality [10].

Fig 1 integrates both the EMV and Intersectionality theories. In the sub-Saharan Africa (SSA) contexts, IPV is conceptualised as both a multidimensional social phenomenon, and an experience shaped by intersecting vulnerabilities. Whereas the EMV identifies specific IPV and CMDs risks and/or protective factors, intersectionality explains how these factors work collectively to position a woman to experience IPV. Thus, IPV among women in SSA is a product of interacting personal, relational, community and societal influences.

**Fig 1:**
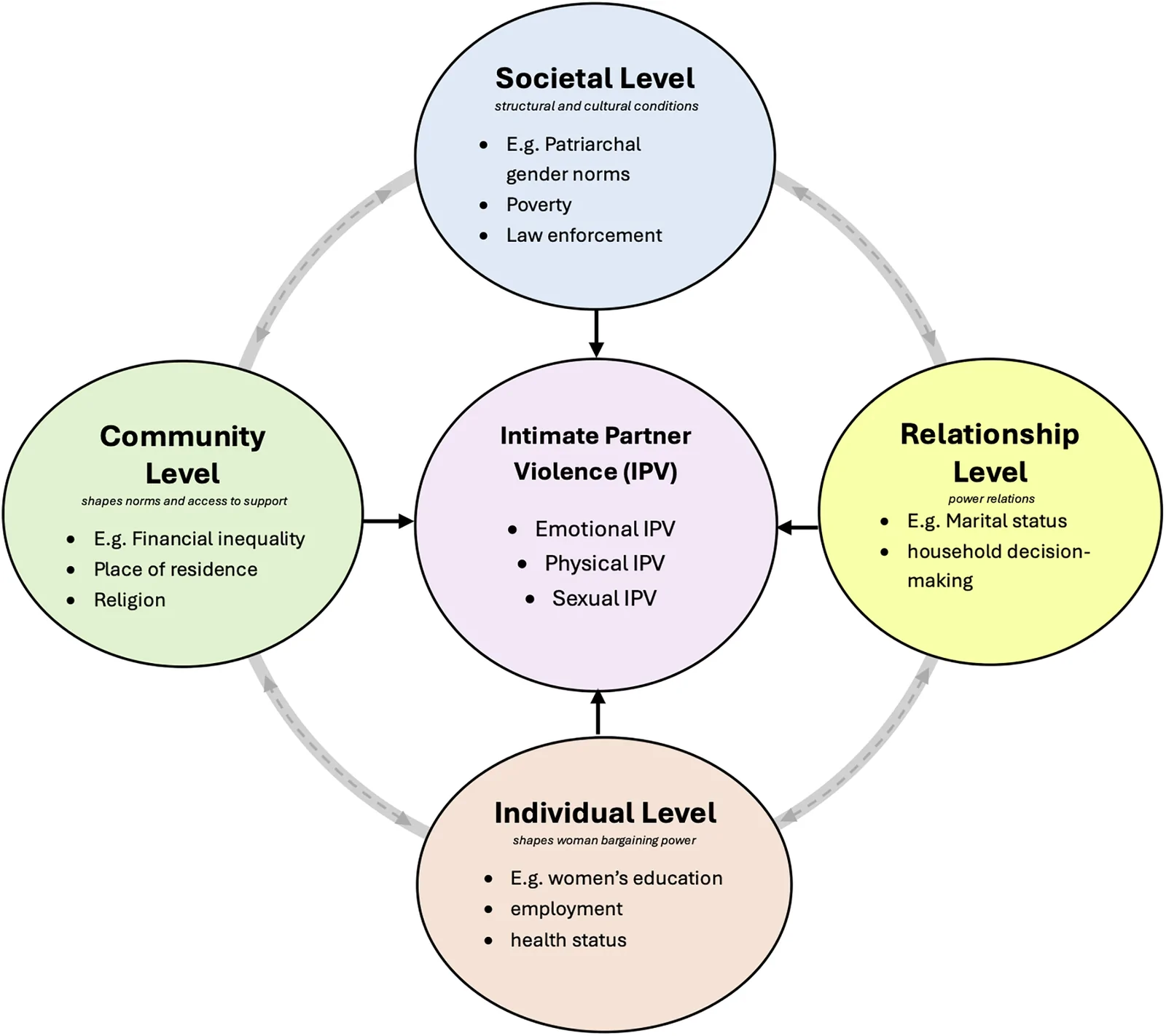
Intersectionality of the Multidimensional Determinants of Intimate Partner Violence in LMICs. Owusu & Weobong (2026), *adapted from West, Cottman, & Gardner (2021)* [19].

Mozambique is one of the few countries in SSA which, for the first time in 2022–2023, introduced the full depression and anxiety modules in its Demographic and Health Survey (MDHS). Using the MZDHS, previous studies have examined the association between IPV and mental health outcomes [1,2]. However, these studies examined the independent association between depression, anxiety and IPV, and used a contextual GAD-7 cut-off point of ≥6 to indicate probable anxiety. Although, this approach helps to examine disorder-specific conditions, depression and anxiety are both CMDs, and often co-occur, share common risk factors, consequences, or interventions in population-based studies [12]. Again, although, the use of different thresholds in these studies may be contextually relevant, the reduced cut-off points may limit international comparability of the results.

Conversely, we note that IPV survivors typically experience mixed anxiety-depressive disorders (MADD), which is a comorbid presentation of anxiety and depression, with evidence demonstrating that sexual, physical, and psychological IPV’s produce mental health effects that are better captured through integrated assessment [12,17]. In SSA, MADD is pronounced due to intersecting social, economic, and structural vulnerabilities. Yet, anxiety and depression are often conceptualised as distinct diagnostic conditions despite their frequent co-occurrence in population-based studies. The association between IPV and mental health is generally consistent and positive, but the magnitude of the association varies across IPV types, past and/or recent exposures, specific mental health outcomes, and population subgroups. Against this background, this study sought to explore a) the association between IPV and CMDs, b) timing (past and/or recent) of IPV and CMDs, and c) association between recent type-specific IPV and CMDs. Further, it empirically assessed one of Intersectionality’s central propositions in relation to whether socio-economic characteristics modify women’s IPV outcomes. These approaches will offer insights into IPV timing and mental health, guide IPV advocacy, and shape national decisions regarding IPV and mental health support for women in Mozambique.

## Materials and methods

### Data and sampling

The recent 2022–2023 Mozambique Demographic and Health Survey (MDHS) was used for this study. The MDHS is implemented by the Mozambique National Institute of Statistics (INE), in collaboration with the Ministry of Health with technical support from the DHS Programme. Field work was conducted from 27th July 2022–27th February 2023 [20]. The MZDHS is designed to collect and provide data on various demographic and health related indicators including mental health [20]. The DHS is a standardised survey and uses cross-sectional design to collect regional/provincially representative data. It utilises a two-stage stratified cluster sampling approach by stratifying provinces into rural and urban segments. In stage 1, primary sampling units (PSUs) were selected based on size and independent selection made in each stratum. In stage 2, households in each stratum were selected systematically with equal probability. As shown in Fig 2, the analysis for this study was restricted to women of reproductive age group (15–49) who answered the IPV module and therefore formed the unit of analysis. Women who were eligible to answer the IPV module were those who were in union. All women who were not eligible or had incomplete but missing at random IPV or mental health data were excluded from the study. Weighting was done using the *svyset* function in STATA.

**Fig 2:**
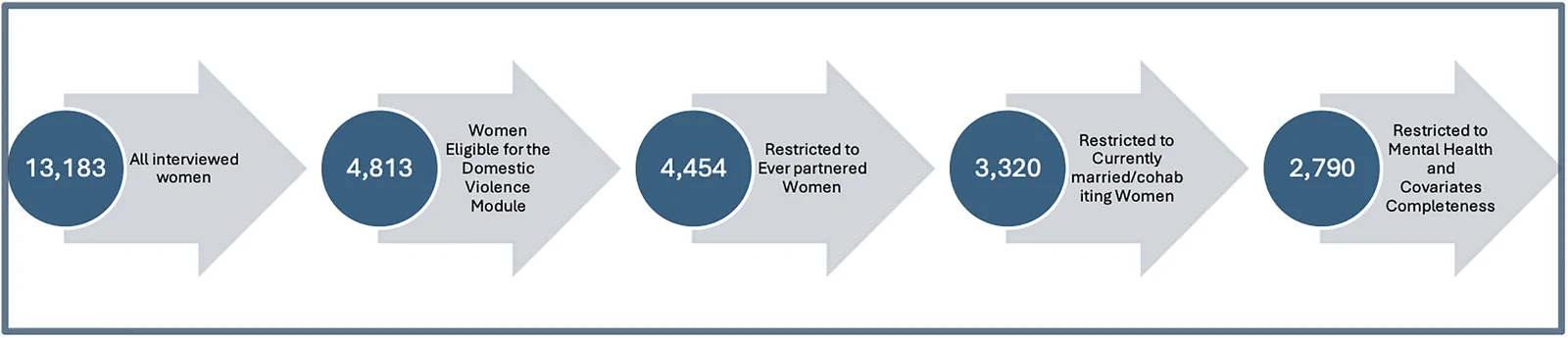
Flow Chart of the Sample Selection.

### Variables

#### Outcome variable.

The main outcome variable for this study is common mental disorders (CMDs) – a composite variable computed from the depression or anxiety variables in the 2022–2023 MDHS. Depressive symptoms were assessed using the 9-item Patient Health Questionnaire (PHQ-9) which was asked of women about their depression status in the two weeks before the day of the survey [21]. PHQ-9 is scored on a 4-point Likert-type scale from 0 (not at all) to 3 (nearly every day). With its total score ranging from 0 to 27; a cut-off score of 10 or higher indicative of probable depression was coded 1, compared to a score below 10 which was coded 0 to indicate non probable depression [21]. Similarly, anxiety symptoms were assessed using the 7-item Generalised Anxiety Disorder (GAD-7) assessment tool. The scores range from 0 to 21, with a cut-off score of 10 or higher indicating probable anxiety [22]. The severity scales of the PHQ-9 range from no/minimal, mild, moderate, moderately severe, and severe symptoms. Also, the GAD-7 range from no, mild, moderate and severe symptoms. Consistent with global mental health studies [23,24], CMDs was therefore created to reflect the experience of either probable depression or probable anxiety, reduce misclassification in population-based studies, and to enhance statistical power and stability of estimates in regression and moderation models. Regarding the GAD-7 score cut-off point, this study used a cut-off score of ≥10 or higher to indicate probable anxiety, consistent with other GAD-7 studies [22].

#### Key independent variables.

The main independent variables for this study were physical IPV, emotional IPV, and sexual IPV, which collectively measures IPV. The IPV module was administered to one randomly selected woman of reproductive age in union. The analysis was restricted to women who answered the domestic violence module. Physical IPV was assessed using seven items capturing experiences of being pushed, slapped, punched with a fist, kicked or dragged, strangled or burned, attacked with a knife or gun, or having one’s hair pulled by a husband or partner. Participants provided a binary response (Yes/No). Experience of Physical IPV was coded as present if any of the responses to the above were a ‘Yes’. Emotional IPV was derived from women who experienced threats of harm by husband/partner, insulted or made to feel bad by husband/partner, humiliated in front of others by husband/partner. Those who experienced any of these acts were coded 1 and if none, coded 0. Using similar coding approaches, sexual IPV was also formed from physical coercion into unwanted sex by husband/partner, other unwanted sexual acts by husband/partner, or perform sexual acts woman did not like. All the IPV responses were “never”, “often”, “sometimes”, “yes, but not in the last 12 months”. The “never” responses were coded as 0, the “often” and “sometimes” responses were coded as 1 “yes, within 12 months/recent”, and the “yes, past 12 months” response was coded as 2 “yes, past 12 months”. This coding approaches allowed separate statistical modelling to be developed for past IPV, recent IPV, and type-specific IPV.

#### Potential confounders.

We took a parsimonious approach to controlling for some important socio-demographic, geographic, economic, and health-related woman factors that have been found to be associated with IPV in related studies. These are women’s age, educational level, marital status, household wealth status, place of residence, religion, employment status, mass media exposure, household decision-making, and pregnancy status [1,2].

Younger women face higher risks of IPV while older women face high risk of and poor mental health [1,2]. Education affects women bargaining power, autonomy and IPV risk; it also predicts mental health outcomes. Marital status affects both exposure likelihood to IPV and affects mental health through conflicts and union dissolution [1]. Lower household wealth is associated with higher IPV risk and poorer mental health [1,2]. Place of residence affects IPV norms, service access, reporting, and influence mental health outcomes. Employment status influences empowerment (affects IPV) and financial stress (mental health). Religious norms may shape gender dynamics and IPV tolerance; religion is also associated with coping styles and mental health. Mass media exposure influences awareness, empowerment and reporting of IPV; it is also linked to mental health. Pregnancy increases IPV risk and independently affects mental health (e.g., antenatal depression). Women with lower household decision making power are vulnerable to IPV and poor mental health due to reduced autonomy, stress, and dependency.

### Statistical analysis

The data was analysed using Stata version 18/MP. The analysis considered the complex survey design of the DHS including the two-stage sampling technique, clustering, and stratification. For this study, the domestic violence weight was applied. The *svy:* command was used to report proportions while keeping the unweighted frequencies. At the descriptive level, the background characteristics of the partnered women were described, followed by the prevalence of poor mental health. The bivariate analysis included the weighted association between IPV and poor mental health, as well as background factors and poor mental health tested at the 95% confidence interval with the Rao-Scott adjusted Chi-square. Multivariable analysis involved performing multiple binary logistic regression models to examine the association between IPV, recent IPV, type-specific IPV, and poor mental health, while adjusting for known confounders. Adjusted odds ratios (aOR) with corresponding confidence intervals and p-values were generated. Significant levels were pegged at 0.05. All models were fitted using the same analytic sample and covariate structure to strengthen results comparability. With a mean variance inflation factor (VIF) = 2.06, no problematic multicollinearity was observed (S3 Table). Sensitivity analyses was performed to compare differences between recent and past risk factors for IPV and poor mental health. Separate interaction models to test whether the association between recent IPV and poor mental health varied across selected interacting socio-economic characteristics were performed on the survey-adjusted logistic regression model. Adjusted predicted probabilities and margins plots were reported to facilitate interpretation of the interaction effects. Wald tests were used to assess the overall statistical significance of each interaction term.

### Ethics

Permission to use the 2022–23 Mozambique DHS dataset was sought from the Measure DHS website. Ethical approval was not needed for the secondary analysis as ICF International and national statistical agencies already met all ethical protocols during primary data collection. Coding and reporting of this research followed good ethical reporting.

## Results

In Table 1, the weighted percentage distribution of sampled women in union (married/cohabiting) by their background characteristics is provided. Regarding IPV, 19% of the women experienced IPV within the 12 months preceding the survey while 12.4% had past experiences. Emotional IPV was the most prevalent (16.1%) followed by physical IPV (12%). Regarding those who have experienced recent IPV, 20.4% experienced CMDs, compared to 10.4% among those who did not experience recent IPV. The average age of the women was 31 years (minimum age 15 and maximum age 49 years). Sixty-six percent of them were from rural areas. In terms of education, most of the women (74%) had attained a primary level or lower education, compared to 25% who had attained secondary or higher levels of education. Regarding wealth status, about 64% of the women were in the middle to richest wealth category. Concerning their marital status, about 64% of the women were cohabiting, compared to 36% who were married. Majority (72%) of the women were Christians. Also, 52% of them were exposed to mass media (at least watched television, listened to radio, read newspaper/magazine). In terms of their employment status, 60% of the women were unemployed, and in terms of their household decision-making ability, 74% jointly made major household decisions with their partners/husbands. Finally on their region/province, majority of the women were from the Nampula (22.5%), Tete (13.3%), and Zambézia (11%) provinces.

**Table 1:** Background Characteristics of Partnered Women in Mozambique.

Background Characteristics	Unweighted n (2,790)	Weighted %
**Age Group**		
15-19	257	9.2
20-24	541	19.4
25-29	579	20.2
30-34	458	14.8
35-39	423	14.9
40-44	303	11.8
45-49	229	9.7
**Place of Residence**		
Urban	1007	33.7
Rural	1783	66.3
**Educational Level**		
No education	762	31.0
Primary	1264	43.2
Secondary	683	23.1
Higher	81	2.7
**Wealth Status**		
Poorest	417	17.4
Poorer	484	18.7
Middle	590	21.1
Richer	615	20.6
Richest	684	22.2
**Marital Status**		
Married	912	36.2
Cohabitation	1,878	63.8
**Religion**		
Christianity	2,032	72.4
Islamic	549	20.3
No religion/other	209	7.3
**Mass Media Exposure**		
No	1,223	47.6
Yes	1,567	52.4
**Employment Status**		
Unemployed	1,504	60.2
Employed	1,273	39.8
**Pregnancy Status**		
Pregnant	281	10.2
Not pregnant	2,509	89.8
**Household Decision-making**		
Yes	520	21.7
Jointly with partner	2,147	74.1
No	123	4.2
**Province**		
Niasa	274	8.0
Cabo Delgado	346	6.6
Nampula	285	22.5
Zambézia	155	11.0
Tete	336	13.3
Manica	202	6.0
Sofala	306	8.7
Inhambane	220	4.7
Gaza	220	4.9
Maputo	253	9.7
Cidade de Maputo	193	4.0
**IPV Occurrence**		
Never	1876	68.5
Within 12 months	544	19.1
Past 12 months	370	12.4
**Emotional IPV**		
Yes	471	16.1
No	2319	83.9
**Physical IPV**		
Yes	350	12.0
No	2440	88.0
**Sexual IPV**		
Yes	139	4.0
No	2651	96.0
**Recent IPV**		
Yes	653	23.4
No	2137	76.6

unweighted n: actual frequencies or sample size.

weighted %: complex survey weighted proportions.

Fig 3 illustrates the weighted distribution of probable depression and anxiety severities among the study population. For depressive symptoms, 72.8% of women reported having no/minimal symptoms, 18.6% showed mild symptoms, 4.8% showed moderate symptoms, 3.1% showed moderately severe symptoms, and 0.7% showed severe symptoms. In all, 8.6% of the women had probable depression (Fig 4). Regarding the severity of anxiety, 67.2% of the women had no symptoms, 22.1% had mild symptoms, 8.4% had moderate symptoms, and 2.3% had severe symptoms. In all, 10.7% of the women had probable anxiety (Fig 4).

**Fig 3:**
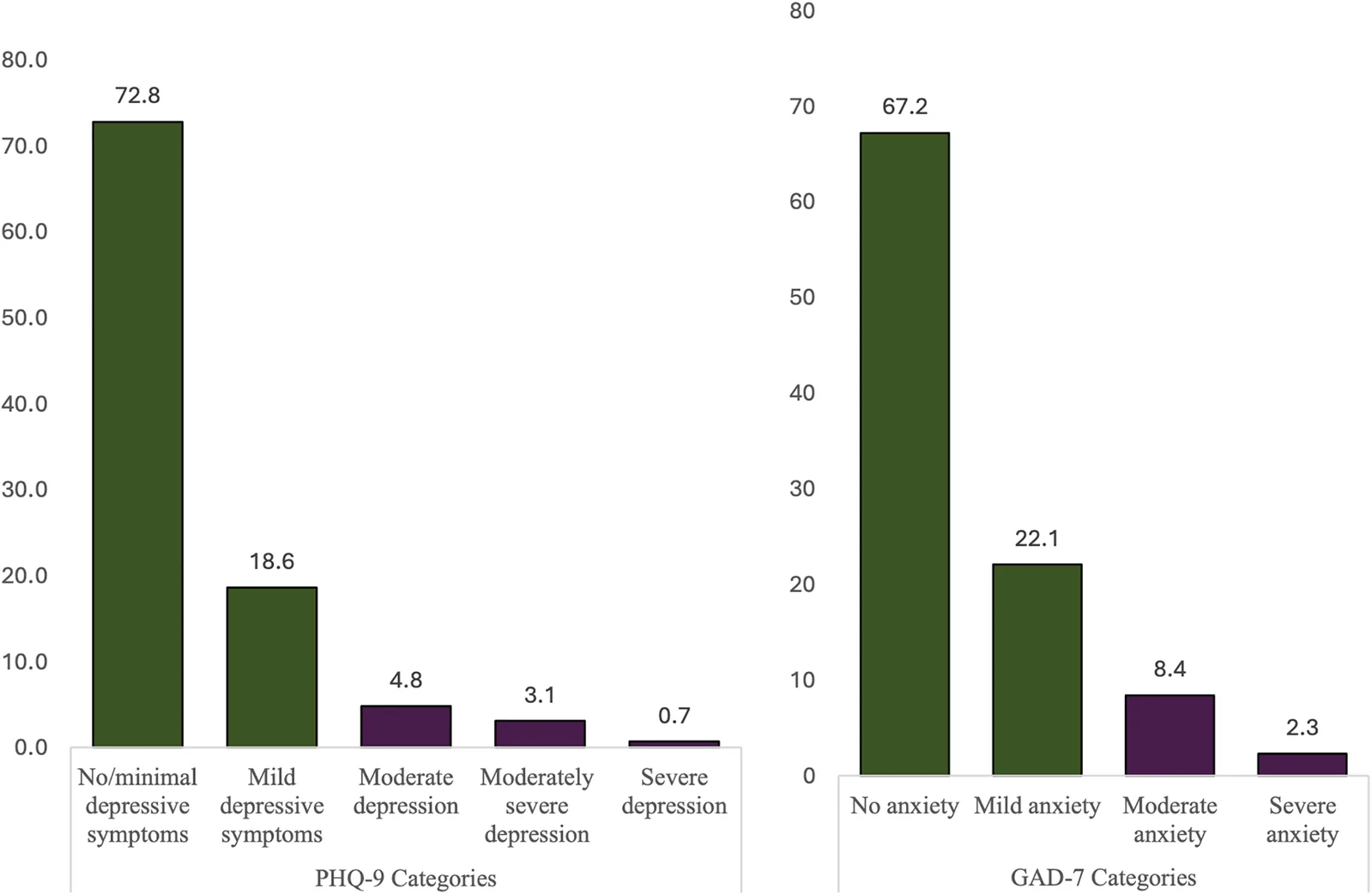
Severity of Depressive and Anxiety Symptoms among Partnered Women in Mozambique.

**Fig 4:**
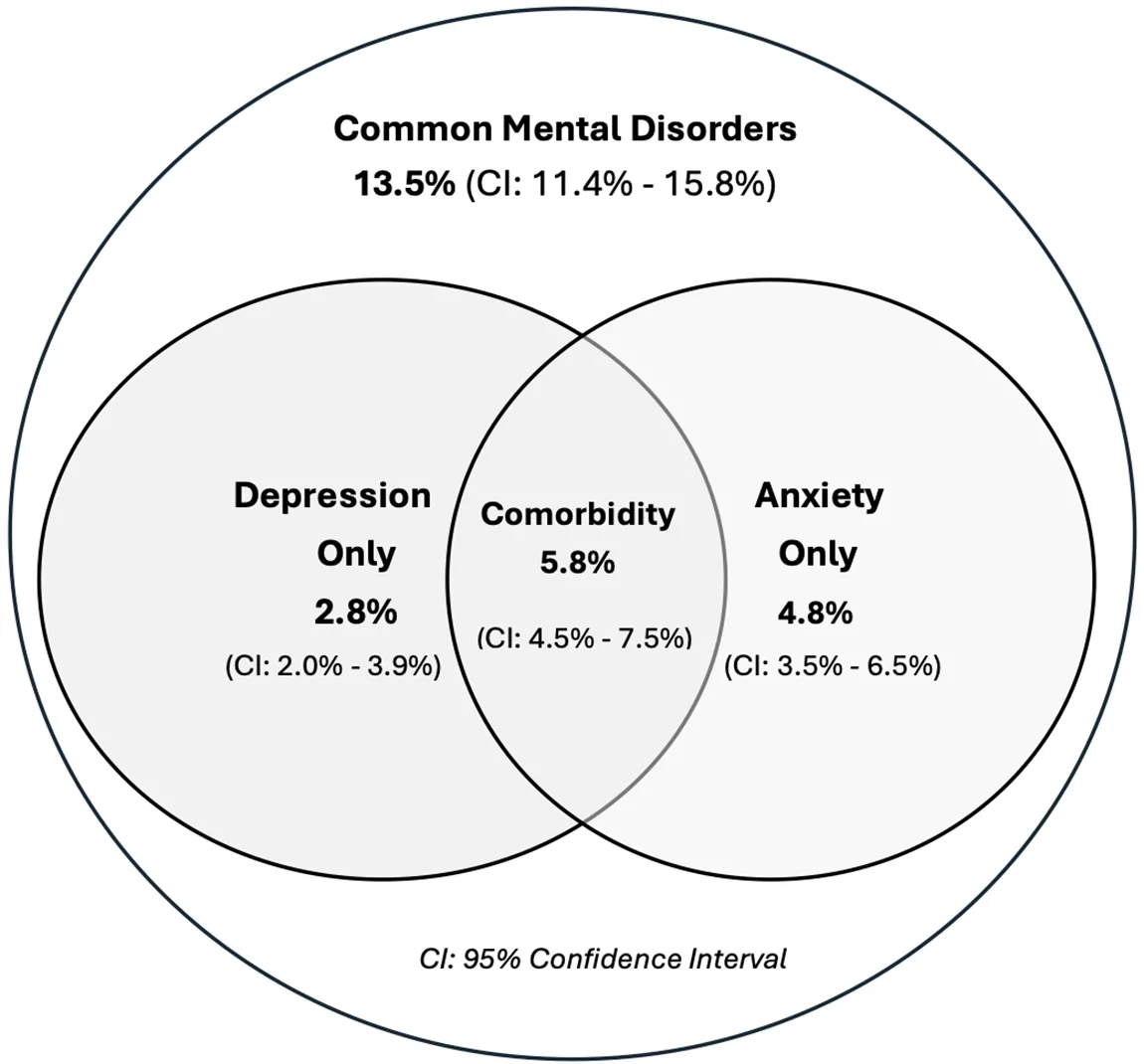
Prevalence of Probable Depression, Anxiety, Comorbidity, and Common Mental Disorders (CMDs) among Partnered Women in Mozambique.

Fig 4 shows that, altogether, 13.5% of the respondents experienced CMDs. Comorbid depression and anxiety were observed among 5.8% of the partnered women (S2 Table).

### Association between Intimate Partner Violence and Mental Health

The weighted percentage distribution of sampled women by their CMD status and background characteristics, corresponding 95% confidence intervals as well as the Rao-Scott adjusted Chi-square are shown in Table 2. The Chi-square test results revealed that the observed differences in CMDs across timing of IPV, physical IPV, emotional IPV, sexual IPV, wealth status, employment status, household decision making, and province/region were significant (α = 0.05). CMD was highest (19.8%) among women who experienced recent IPV, slightly higher (13.8%) among those who experienced IPV in the past 12 months, and lowest (11.6%) among those who have never experienced IPV. Also, 21.1% of women who had experienced physical IPV reported CMDs, compared to 12.4% of those who did not experience physical IPV. A similar observation was observed among women who experienced emotional IPV. Also, 23.3% of women who experienced sexual IPV had CMDs, compared to 13.1% of those who did not experience it.

**Table 2:** Bivariate Association between Intimate Partner Violence and Mental Health.

Respondent Characteristics	Common Mental Disorder (PHQ-9 or GAD-7)		p-value
	No: 86.5% (95% CI: 84.1 – 88.6)	Yes: 13.4% (95% CI: 11.3 – 15.8)	
**Age Group**			0.964
15-19	88.2	11.8	
20-24	87.2	12.8	
25-29	85.0	15.0	
30-34	85.6	14.4	
35-39	87.0	13.0	
40-44	88.2	11.8	
45-49	85.0	15.0	
**Place of Residence**			0.744
Urban	87.0	13.0	
Rural	86.3	13.7	
**Educational Level**			0.249
No education	84.8	15.3	
Primary	86.5	13.5	
Secondary	87.7	12.3	
Higher	97.4	2.6	
**Wealth Status**			0.003**
Poorest	78.8	21.2	
Poorer	86.6	12.4	
Middle	88.7	11.3	
Richer	85.5	14.5	
Richest	91.5	8.5	
**Marital Status**			0.084
Married	84.0	16.0	
Cohabitation	88.0	12.0	
**Religion**			0.057
Christianity	87.4	12.6	
Islamic	81.8	18.2	
No religion/other	91.1	8.9	
**Mass Media Exposure**			0.197
No	84.9	15.1	
Yes	88.0	12.0	
**Employment Status**			0.003**
Unemployed	84.3	15.7	
Employed	90.1	9.9	
**Pregnancy Status**			0.0481**
Pregnant	79.7	20.3	
Not pregnant	87.3	12.7	
**Household Decision-making**			0.044**
Yes	90.9	9.1	
Jointly with partner	85.3	14.7	
No	85.8	14.2	
**Province**			0.000**
Niasa	95.6	4.2	
Cabo Delgado	85.6	14.4	
Nampula	70.7	29.3	
Zambézia	83.4	16.6	
Tete	92.7	7.3	
Manica	96.6	3.4	
Sofala	88.9	11.1	
Inhambane	93.8	6.2	
Gaza	99.3	0.7	
Maputo	89.8	10.2	
Cidade de Maputo	94.0	6.0	
**IPV Occurrence**			0.005**
Never	88.4	11.6	
Within 12 months	80.2	19.8	
Past 12 months	86.2	13.8	
**Emotional IPV**			0.000**
Yes	77.4	22.6	
No	88.3	11.7	
**Physical IPV**			0.002**
Yes	78.9	21.1	
No	87.6	12.4	
**Sexual IPV**			0.004**
Yes	76.7	23.3	
No	86.9	13.1	
**Recent IPV**			0.000**
Yes	79.6	20.4	
No	89.2	10.8	

CI – confidence interval, IPV – Intimate partner violence, **p < 0.005.

Furthermore, women from the poorest households had a significantly higher proportion of CMDs (21.2%), compared to those from richest households (8.5%). Also, women who were pregnant showed higher prevalence of CMDs (20.3%) compared to those who were not pregnant or unsure (12.7%). Likewise, women who made joint household decision with their partners had a higher proportion of CMDs (14.7%), compared to those who made decisions alone (9.1%). Women who were employed had a significantly lower proportion of CMDs (9.9%) compared to those who were unemployed (15.7%). The prevalence of CMDs varied significantly across provinces, ranging from 0.7% in Gaza to 29.3% in Nampula. Higher prevalence was observed in Nampula (29.3%), Zambézia (16.6%), and Cabo Delgado (14.4%), while lower prevalence was recorded in Gaza (0.7%), Manica (3.4%), and Niassa (4.2%). There was no significant association between women’s age, place of residence, education, marital status, religion, and mass media exposure.

### Determinants of CMDs among partnered women in Mozambique

In the unadjusted relationship (S1 Table), women who experienced any IPV within the past 12 months had higher odds of CMDs compared to those with no IPV (OR = 1.86, 95% CI: 1.28–2.72). Although, those who experienced any IPV in the past 12 months had higher odds of CMDs compared to those who did not experience any IPV (OR = 1.21, 95% CI: 0.73–2.0), this was not statistically significant. The adjusted Wald test indicated that the association for any IPV within the past 12 months was significantly stronger than that for past 12 months (F = 11.86, p = 0.006). After adjusting for socio-demographic, geographic, economic, decision-making, and pregnancy status among women who experienced recent IPV as shown in Fig 5 and S1 Table, recent IPV experience remained significant, showing more than twice the odds of poor mental health (aOR = 2.29, 95% CI: 1.55 – 3.39), compared to those who did not experience recent IPV (past 12 months and never).

**Fig 5:**
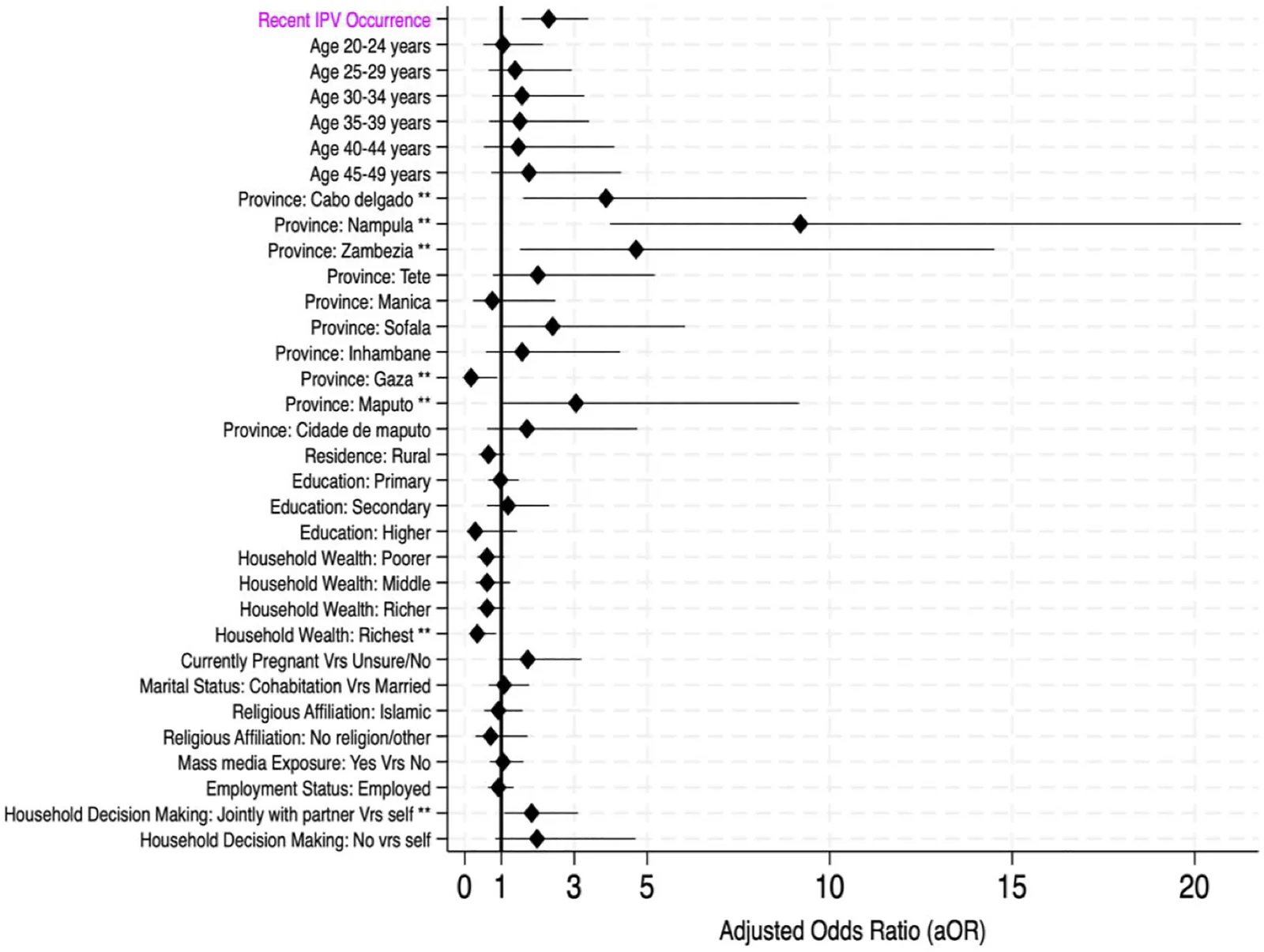
Adjusted Odds Ratios of CMDs by Recent IPV Occurrence among Partnered Women in Mozambique.

In terms of wealth status, women from the richest households had a significantly lower odds of CMDs (aOR =0.34, 95% CI: 0.13 – 0.85), compared to those from poorest households. Lower household decision making autonomy was also independently associated with higher odds of CMDs (aOR = 1.83, 95% CI: 1.08 – 3.10), compared to those with higher decision-making autonomy. There was a geographic heterogeneity with CMDs. Women who resided in Nampula (aOR=9.19, 95% CI: 3.97 – 21.26), Cabo Delgado (aOR=3.87, 95% CI: 1.59 – 9.36), Zambézia (aOR = 4.69, 95% CI: 1.51 – 14.51), and Maputo (aOR=3.04, 95% CI: 1.01 – 9.16), were greater than three folds more likely to have CMDs, compared to women who resided in Niasa. The sensitivity test of association between IPV and CMDs produced stable associative factors as shown in S1 Table.

#### Recent type-specific IPV and CMDs.

As shown in Table 3, in the crude association, women who experienced physical IPV (OR = 1.89, 95% CI: 1.25–2.84), emotional IPV (OR = 2.20, 95% CI: 1.59–3.05), and sexual IPV (OR = 2.03, 95% CI: 1.22–3.35), had a significantly higher odds of CMDs compared to those who did not experience IPV. After adjusting for the same covariates in Fig 5 and S1 Table, women who experienced emotional IPV remained statistically significant, having double the odds of CMDs (aOR = 2.34, 95% CI: 1.59 – 3.44).

**Table 3:** Association between Recent Type-Specific IPV and CMDs (Model 3).

Background Characteristics	OR	95% CI	p-value
**Crude Association**			
Physical IPV	1.89	1.25 – 2.84	0.002**
Emotional IPV	2.20	1.59 – 3.05	0.000**
Sexual IPV	2.03	1.22 – 3.35	0.006**
**OR (adjusted odds ratios)**			
**Physical IPV**			
No	Ref		
Yes	1.21	0.73 - 2.03	0.448
**Emotional IPV**			
No	Ref		
Yes	2.34	1.59 - 3.44	0.000**
**Sexual IPV**			
No	Ref		
Yes	1.67	0.83 - 3.35	0.150
**Age Group**			
15-19	Ref		
20-24	1.05	0.50 - 2.19	0.890
25-29	1.34	0.62 - 2.91	0.448
30-34	1.55	0.73 - 3.27	0.249
35-39	1.46	0.63 - 3.39	0.373
40-44	1.48	0.52 - 4.17	0.452
45-49	1.76	0.71 - 4.33	0.217
**Place of Residence**			
Urban	Ref		
Rural	0.64	0.38 - 1.07	0.095
**Educational Level**			
No education	Ref		
Primary	0.95	0.62 - 1.45	0.826
Secondary	1.17	0.59 - 2.30	0.644
Higher	0.29	0.06 - 1.44	0.133
**Wealth Status**			
Poorest	Ref		
Poorer	0.63	0.36 - 1.11	0.115
Middle	0.63	0.31 - 1.28	0.203
Richer	0.63	0.35 - 1.11	0.112
Richest	0.34	0.13 - 0.88	0.026**
**Marital Status**			
Married	Ref		
Cohabitation	1.05	0.65 - 1.72	0.815
**Religion**			
Christianity	Ref		
Islamic	0.93	0.54 - 1.58	0.793
No religion/other	0.70	0.28 - 1.69	0.429
**Mass Media Exposure**			
No	Ref		
Yes	1.02	0.66 - 1.57	0.925
**Employment Status**			
Unemployed	Ref		
Employed	0.93	0.64 - 1.37	0.740
**Pregnancy Status**			
Not pregnant/unsure	Ref		
Pregnant	1.72	0.93 - 3.19	0.081
**Household Decision-making**			
Yes	Ref		
Jointly with partner	1.84	1.08 - 3.15	0.025**
No	2.04	0.84 - 4.93	0.111
**Province**			
Niasa	Ref		
Cabo Delgado	3.95	1.65 - 9.46	0.002**
Nampula	9.56	4.15 - 22.03	0.000**
Zambézia	4.86	1.59 - 14.81	0.005**
Tete	2.02	0.78 - 5.23	0.143
Manica	0.74	0.23 - 2.42	0.630
Sofala	2.38	0.95 - 5.92	0.061
Inhambane	1.61	0.60 - 4.31	0.343
Gaza	0.17	0.03 - 0.90	0.038**
Maputo	3.03	1.01 - 9.05	0.046**
Cidade de Maputo	1.69	0.61 - 4.66	0.304

Ref: Reference category.

### Does the association between recent IPV and CMDs differ across Women’s intersecting socio-economic characteristics: Test of Intersectionality theory

Separate interaction models were estimated to assess whether the association between IPV and CMDs differed across women’s socio-economic positions. As shown in Table 4, interaction analyses using adjusted predicted probabilities indicated that marital status marginally modified the association between IPV and CMDs (Wald p = 0.038). Among married women, the predicted probability of CMDs increased modestly from 12.0% among those without recent IPV to 15.6% among those with recent IPV. In contrast, among cohabiting women, the predicted probability increased substantially from 10.6% to 24.5%. Although, the richer wealth quintile was statistically significant, the joint Wald test showed no evidence of interaction effect between IPV and wealth status on CMD. In addition to household wealth, no statistically significant interaction effects were observed for educational level, place of residence, employment status, or household decision-making although the margins plot showed consistently higher predicted probabilities of CMDs among women who experienced recent IPV across all categories of the variables as shown in Fig 6.

**Table 4:** Adjusted Predicted Probabilities of CMDs by Recent IPV Exposure across Intersecting Socio-economic Positions of Partnered Women in Mozambique.

Intersecting Variables	Adjusted Predicted Probability		Wald p-value	Recent IPV [95% CI]
	No IPV (%)	Recent IPV (%)		
**IPV##Household Wealth**			0.871	
Poorest	18.8	21.6		10.8 – 32.3
Poorer	10.8	22.8		11.6 – 34.1
Middle	11.4	20.7		8.0 – 33.5
Richer*	9.6	26.6		17.8 – 35.4
Richest	6.7	13.9		5.3 – 22.5
**IPV##Educational Level**			0.393	
No education	11.3	20.0		11.7 – 28.3
Primary	11.0	20.3		14.1 – 26.6
Secondary	12.2	25.2		14.6 – 35.9
Higher	4.6	N/A		
**IPV##Place of Residence**			0.407	
Urban	13.5	27.8		18.5 – 37.1
Rural	10.4	18.0		12.7 – 23.3
**IPV##Marital Status**			0.038*	
Married	12.0	15.6		8.5 – 22.8
Cohabitation	10.6	24.5		18.9 – 30.1
**IPV##Employment Status**			0.085	
Unemployed	12.1	18.7		13.1 – 24.3
Employed	9.3	23.9		17.1 – 30.8
**IPV##Household Decision-making**			0.576	
Yes	6.6	18.1		8.9 – 27.4
Jointly with partner	12.5	21.7		16.3 – 27.2
No	13.1	23.7		8.4 –39.0

*p-value <0.05. The richer category shows an individual interaction coefficient significance.

N/A- unstable estimate due to smaller sample size.

Predicted probabilities were estimated from survey-adjusted logistic regression models Wald p-values represent the overall significance of the interaction terms.

**Fig 6:**
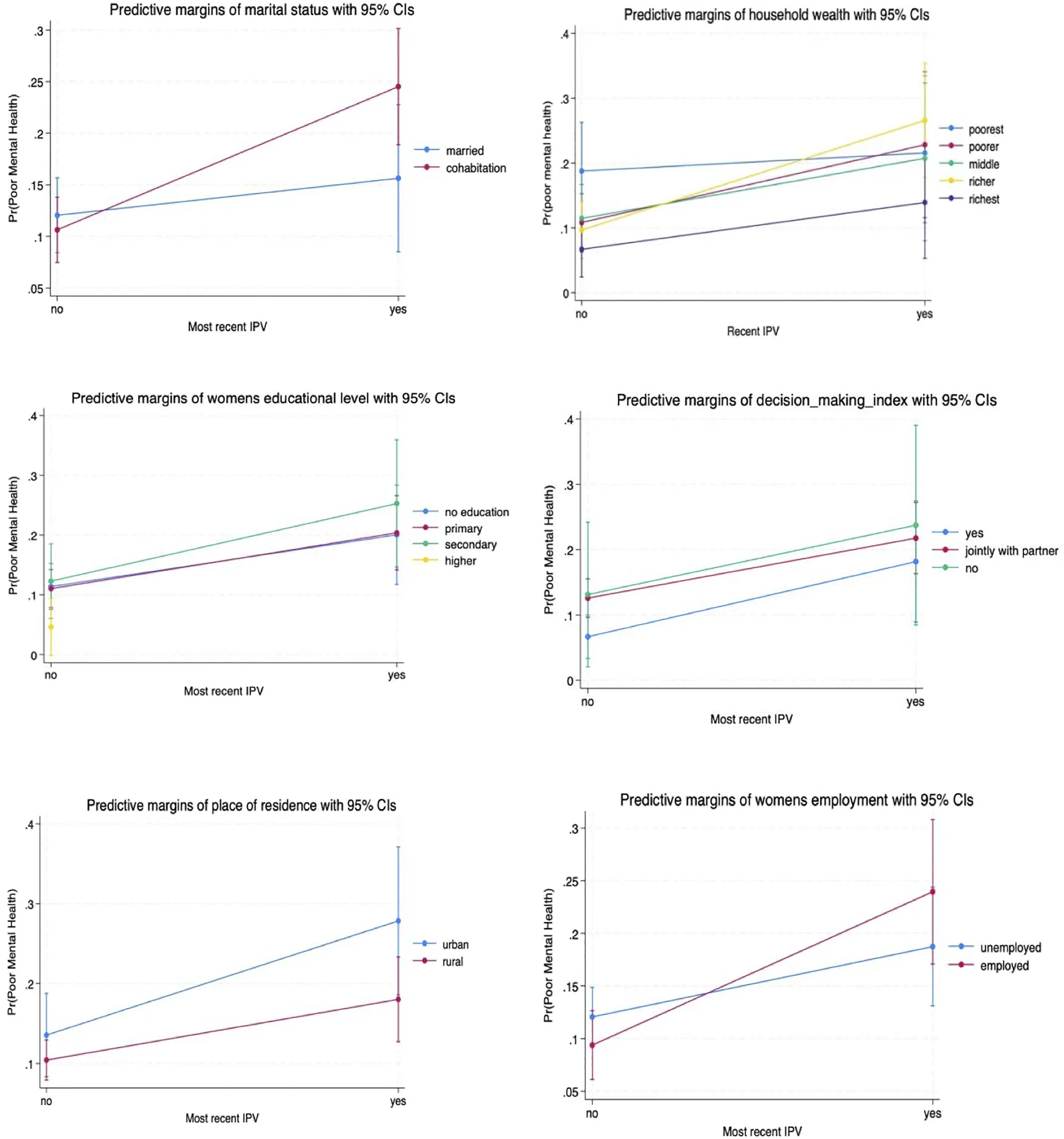
Margins Plot of Predicted Probabilities of IPV and Intersecting Social Position Variables.

## Discussion

This study examined the association between intimate partner violence (IPV) and common mental disorders (CMDs) among women in union in Mozambique. The results show that 13.5% of the women exhibited symptoms of CMDs, including probable depression (8.6%) and anxiety (10.7%). Recent IPV (i.e., IPV within the past 12 months) was independently and significantly associated with CMDs, while IPV occurring in the past 12 months prior showed elevated but not statistically significant odds. Emotional IPV emerged as the only type-specific IPV form independently associated with CMDs after adjustment for confounders.

The strong association between recent IPV and CMDs aligns with a robust body of evidence demonstrating that exposure to IPV increases women’s risk of CMDs [1–3]. The temporal pattern where recent IPV shows a stronger and statistically significant association compared to past IPV, supports the evidence that the psychological impact of IPV may be more acute in the immediate aftermath of exposure, aligning with broader evidence suggesting the temporal proximity of IPV exposure on mental health [25–27]. While past IPV remained positively associated with CMD, the lack of statistical significance may suggest attenuation over time, resilience processes, learned helplessness, or insufficient statistical power to detect smaller effects [3,26,28].

The independent association between emotional IPV and CMDs is particularly noteworthy. It highlights the profound psychological harm inflicted by emotional abuse beyond physical or sexual violence. Emotional abuse has increasingly been recognised as a strong determinant of psychological distress, sometimes with effects comparable to or exceeding those of physical or sexual IPV [29,30]. Emotional IPV may undermine self-worth, autonomy, and social connectedness, thereby contributing directly to poor mental health. Further, emotional IPV is often more chronic, and has persistent psychological effects, or direct impact on mood and thoughts, compared to physical violence which may have episodic occurrence and strong immediate trauma, but less continuous psychological impact [31,32]. Given its high prevalence, emotional abuse functions as a key driver of internalised oppression. From an intersectional perspective, this form of abuse directly targets victims intersecting identities, thereby stripping away their sense of agency [10]. Noting that emotional abuse is often overlooked, this finding reinforces the need to treat emotional IPV not as a secondary or less severe form of violence, but as a critical issue of public mental health concern.

At the individual level, factors such as a woman’s age, education, health status among others shape vulnerability to IPV by influencing awareness, autonomy, and economic dependence [1,2]. Economic resources may buffer against psychological distress by increasing access to health care, social support, and coping resources [33,34]. This finding echoes prior research linking poverty and economic stress to both IPV exposure and CMD outcomes [1]. Wealthier women’s lower odds of CMDs reflects findings from multi-country analyses suggesting complex and context-dependent relationships between socioeconomic status and IPV, with Mozambique uniquely showing higher IPV odds even among the richest groups [35]. In line with intersectionality, as women from low-income background experience compounded vulnerability of IPV, the trauma from the abuse and stress from material deprivation limits their ability to leave such partnerships, as financial dependence is often weaponised by the perpetrators in the form of economic coercion [10]. This complexity underscores the importance of recognizing that IPV and CMDs consequences transcend economic boundaries, requiring broad-based prevention strategies incorporating economic, social, and gender equity considerations.

Additionally, although, joint household decision-making is often conceptualized as a marker of empowerment, the finding that women involved in joint household decision-making have higher odds of CMDs compared to those making decisions alone may reflect underlying relational dynamics or stressors not captured in this analysis. Broader structural conditions including patriarchy and subordinate role of women in society may expose them to IPV [18]. In SSA contexts where gender norms are shifting, shared decision-making may co-exist with tension/conflict, reduced autonomy, or coercive control, potentially contributing to psychological stress. Within this perspective, joint household decision making may not always mean harmonious decision making. As such, in cultures where men try to maintain traditional power structures, a woman’s attempt to assert agency may trigger a backlash of psychological control [10]. This could also be due to decision-making burden where joint decisions may increase women’s cognitive and emotional workload especially if they remain primary for implementing them. Also, it could be due to selection effects, where partnered women facing financial hardships, illness, or other stressors (directly associated with CMDs) may adopt joint decision-making as a form of coping with the stressors of life.

Geographical disparities in CMDs across provinces such as Nampula, Cabo Delgado, Zambezia, Gaza, and Maputo suggest regional variations in IPV prevalence, highlighting the importance of contextual vulnerability. Regional inequities, conflict exposure (particularly in Cabo Delgado), socioeconomic deprivation, cultural norms, and differential access to services may all shape mental health outcomes. In particular, Cabo Delgado has experienced prolonged armed conflict and displacement, conditions that may exacerbate trauma, dominance, and violence among women in the region [36,37]. The higher odds of CMDs in Maputo (capital city) may be linked to urban stressors, economic inequalities, and greater awareness and reporting of mental health problems among women in the region [36]. Conversely, women in rural areas are less likely to report CMDs compared to urban women [36]. Hence, the lower odds of CMDs in Gaza may reflect relatively lower stress exposure, stronger social support systems, or differences in reporting and help-seeking behaviours. However, the wide confidence intervals (reflecting smaller sample size and high variability), indicate imprecise estimates. Although, the statistical significance of province suggests a robust association, we caution the interpretation of the association between province and CMDs. For example, in some provinces, partnered women are 9 times more likely to have CMDs compared with women in Niasa, the confidence intervals show that this likelihood could be as reduced as 4 or as high as 21.

Intersectionality provides a deeper understanding of how social identities including wealth, education, residence, marital status, employment, and household decision making operating at varying individual, relational, community, and societal levels overlap to influence IPV. Rather than examining these identities separately, intersectionality emphasises their synergistic effect in producing outcomes. The results indicate that the association between IPV and CMDs among partnered women is significantly modified by marital status. Cohabiting women’s greater risks of psychological vulnerability following IPV may reflect weaker social protection, relationship instability, or limited support systems, compared to their married counterparts. For instance, financial dependence, restricted autonomy, and socio-cultural barriers that hinder help-seeking and exiting abusive relationships are pronounced among cohabiting women [2,38,39]. While intersectionality theory offers a useful framework for understanding how multiple social positions interact to influence CMDs, the results provide partial empirical support for intersectionality by demonstrating that certain socio-economic positions, particularly marital contexts, shape women’s psychological vulnerability to IPV.

### Policy and programmatic implications, and recommendations for research

Given the strong association between recent IPV and CMDs, integrating routine IPV screening into primary health care and reproductive health services is critical. Scaling up community-based mental health services within primary care, consistent with global mental health recommendations [40,41], could improve access to care for women experiencing IPV-related distress. Second, emotional IPV must be explicitly addressed in national gender-based violence policies and programming, implying that prevention campaigns and service provider training should emphasize the psychological harms of non-physical abuse. Third, social protection and poverty reduction strategies may have secondary mental health benefits. Consequently, strengthening economic empowerment initiatives for women, while ensuring they are coupled with gender-transformative information may reduce vulnerability to both IPV and CMDs [42–44].

In terms of further research, the results warrant mixed-methods exploration to elucidate the mechanisms linking household power dynamics to women’s mental health outcomes. Also, there is the need to gauge the precise regional variations and its predictors to warrant targeted mental health interventions. Again, future studies could apply the conceptual framework in this study to examine how intersectionality, especially from a structural perspective shape partnered women’s experiences of IPV and CMDs.

### Strengths and limitations

The sampling approach used for the DHS enabled sample weighting to produce estimates that are nationally, regionally, and urban/rural representative. Standardized measures and thresholds of the outcome variable from the DHS was used. Unlike previous studies, this study foreground both the Ecological Model of Violence and the Intersectionality Theory to provide a multidimensional understanding of IPV and CMDs. The study further extends conventional regression approaches by incorporating interaction analysis and adjusted predicted probabilities to examine intersectional variations in the mental health burden of IPV.

Despite the strengths of this study, its cross-sectional design does not warrant causal inference. Combining depression and anxiety into a single CMD outcome implies that the results reflect associations with general psychological distress (common mental disorders) rather than disorder-specific effects. Also, IPV and CMDs were self-reported, raising the possibility of underreporting due to stigma or fear. The observed stronger association between IPV and CMDs compared with past IPV may reflect differential reporting or recall patterns and should therefore be interpreted with caution. Additionally, residual confounding cannot be excluded. Again, the measures of CMDs are not diagnostic tools, and raises concerns about cultural validity. The interaction terms which were not statistically significant may reflect limited statistical power within sub-groups. The study did not capture broader structural dimensions of intersectionality such as social norms or community level gender relations and disability, which are key limitations of using secondary data. Also, the study focused on women in union, and not all women aged 15–49 years. Despite these limitations, the study provides important evidence about the association between IPV and poor mental health among women in union in Mozambique.

## Conclusion

This study adds to the growing evidence of intimate partner violence's (IPV’s) detrimental association with women’s mental health in Mozambique, highlighting that recent IPV is particularly salient for poor mental health outcomes, while also showing that emotional IPV plays a key role alongside physical and sexual violence. The findings further underscore the importance of applying intersectional lens to understand the role of IPV on mental health. Economic disadvantage and regional disparities further compound poor mental health risk. Addressing IPV must be a public health priority, integrating mental health care with violence prevention and socioeconomic interventions tailored to the country’s unique social and geographic context. Such multi-sectoral approaches will be essential to improving the wellbeing and safety of partnered women in Mozambique.

## Supporting information

S1 Table Association between IPV Occurrence, Recent IPV Occurrence, and Poor Mental Health among Partnered Women in Mozambique. (DOCX)

S2 Table Weighted Percentage of Depression and Anxiety Comorbidity. (DOCX)

S3 Table Multicollinearity Test of the Study Variables. (DOCX)

S1 Fig Graphical Abstract. (TIFF)
