# Supplementary material for: Intimate partner violence and common mental disorders among women in Mozambique: Evidence from the 2022-2023 Mozambique Demographic and Health Survey
Source: PLOS Ment Health. 2026 Oct 13;3(10):e0000660. doi: 10.1371/journal.pmen.0000660 (PMC13660890; doi:10.1371/journal.pmen.0000660)
Supplement: Supplementary file 1 [file pmen.0000660.s001.docx]

**S1 Table.** Association between IPV Occurrence, Recent IPV Occurrence, and Poor Mental Health among Partnered Women in Mozambique

| Predictors | Model 1: Poor Mental Health (IPV Occurrence) | | Model 2: Poor Mental Health (Recent IPV) | |
| --- | --- | --- | --- | --- |
|  | OR (95% CI) | P-value | OR (95% CI) | P-value |
| **Crude Association** |  |  |  |  |
| **IPV Occurrence** |  |  |  |  |
| No | Ref |  |  |  |
| Yes, within 12 months (recent) | 1.86 (1.28–2.72) | 0.001** | 2.01 (1.42–2.84) | 0.000*** |
| Yes, past 12 months | 1.21 (0.73–2.00) | 0.456 | ––––– | ––––– |
|  |  |  |  |  |
| **Adjusted Association** | | | |  |
| **IPV Occurrence** |  |  |  |  |
| No | Ref |  | Ref**+** |  |
| Yes, within 12 months | 2.05 (1.36 – 3.09) | 0.001** | 2.29 (1.55 – 3.39) | 0.000*** |
| Yes, past 12 months | 1.40 (0.80 – 2.44) | 0.237 |  |  |
|  |  |  |  |  |
| **Age Group** |  |  |  |  |
| 15-19 | Ref |  | Ref |  |
| 20-24 | 1.06 (0.52 – 2.17) | 0.861 | 1.04 (0.50 – 2.13) | 0.907 |
| 25-29 | 1.36 (0.64 – 2.91) | 0.415 | 1.37 (0.64 – 2.92) | 0.402 |
| 30-34 | 1.58 (0.75 – 3.31) | 0.224 | 1.56 (0.74 – 3.27) | 0.232 |
| 35-39 | 1.47 (0.64 – 3.35) | 0.357 | 1.50 (0.66 – 3.40) | 0.325 |
| 40-44 | 1.43 (0.51 – 4.02) | 0.487 | 1.46 (0.52 – 4.09) | 0.462 |
| 45-49 | 1.69 (0.68 – 4.15) | 0.252 | 1.75 (0.72 – 4.28) | 0.214 |
|  |  |  |  |  |
| **Place of Residence** |  |  |  |  |
| Urban | Ref |  | Ref |  |
| Rural | 0.65 (0.39 – 1.09) | 0.107 | 0.64 (0.39 – 1.07) | 0.096 |
|  |  |  |  |  |
| **Educational Level** |  |  |  |  |
| No education | Ref |  | Ref |  |
| Primary | 0.96 (0.63 – 1.46) | 0.865 | 0.97 (0.64 – 1.47) | 0.911 |
| Secondary | 1.15 (0.59 – 2.24) | 0.662 | 1.18 (0.60 – 2.30) | 0.617 |
| Higher | 0.27 (0.55 – 1.38) | 0.118 | 0.28 (0.05 – 1.42) | 0.128 |
|  |  |  |  |  |
| **Wealth Status** |  |  |  |  |
| Poorest | Ref |  | Ref |  |
| Poorer | 0.60 (0.34 – 1.07) | 0.086 | 0.61 (0.34 – 1.07) | 0.087 |
| Middle | 0.60 (0.30 – 1.22) | 0.164 | 0.61 (0.30 – 1.23) | 0.172 |
| Richer | 0.60 (0.34 – 1.06) | 0.082 | 0.61 (0.34 – 1.06) | 0.084 |
| Richest | 0.34 (0.13 – 0.83) | 0.019** | 0.34 (0.13 – 0.85) | 0.022** |
|  |  |  |  |  |
| **Marital Status** |  |  |  |  |
| Married | Ref |  | Ref |  |
| Cohabitation | 1.08 (0.66 – 1.77) | 0.731 | 1.07 (0.65 – 1.75) | 0.776 |
|  |  |  |  |  |
| **Religion** |  |  |  |  |
| Christianity | Ref |  | Ref |  |
| Islamic | 0.91 (0.53 – 1.54) | 0.733 | 0.92 (0.53 – 1.58) | 0.764 |
| No religion/other | 0.74 (0.31 – 1.78) | 0.506 | 0.70 (0.29 – 1.71) | 0.445 |
|  |  |  |  |  |
| **Mass Media Exposure** |  |  |  |  |
| No | Ref |  | Ref |  |
| Yes | 1.03 (0.67 – 1.58) | 0.869 | 1.04 (0.68 – 1.60) | 0.837 |
|  |  |  |  |  |
| **Employment Status** |  |  |  |  |
| Unemployed | Ref |  | Ref |  |
| Employed | 0.91 (0.63 – 1.34) | 0.662 | 0.92 (0.63 – 1.33) | 0.671 |
|  |  |  |  |  |
| **Pregnancy Status** |  |  |  |  |
| Not pregnant/unsure | Ref |  | Ref |  |
| Pregnant | 1.70 (0.92 – 3.12) | 0.086 | 1.72 (0.93 – 3.19) | 0.083 |
|  |  |  |  |  |
| **Household Decision-making** |  |  |  |  |
| Yes | Ref |  | Ref |  |
| Jointly with partner | 1.85 (1.10 – 3.11) | 0.020** | 1.83 (1.08 – 3.10) | 0.024** |
| No | 2.07 (0.89 – 4.77) | 0.088 | 1.97 (0.83 – 4.67) | 0.119 |
|  |  |  |  |  |
| **Province** |  |  |  |  |
| Niasa | Ref |  | Ref |  |
| Cabo Delgado | 3.86 (1.54 – 9.65) | 0.004** | 3.87 (1.59 – 9.36) | 0.003** |
| Nampula | 9.15 (3.93 – 21.29) | 0.000*** | 9.19 (3.97 – 21.26) | 0.000*** |
| Zambézia | 4.74 (1.52 – 14.77) | 0.007** | 4.69 (1.51 – 14.51) | 0.007** |
| Tete | 2.01 (0.76 – 5.31) | 0.157 | 2.00 (0.76 – 5.20) | 0.155 |
| Manica | 0.78 (0.24 – 2.57) | 0.694 | 0.75 (0.22 – 2.47) | 0.640 |
| Sofala | 2.45 (0.96 – 6.22) | 0.060 | 2.40 (0.96 – 6.03) | 0.061 |
| Inhambane | 1.56 (0.57 – 4.24) | 0.380 | 1.57 (0.58 – 4.25) | 0.373 |
| Gaza | 0.17 (0.03 – 0.89) | 0.037** | 0.17 (0.33 – 0.88) | 0.035** |
| Maputo | 3.09 (1.01 – 9.42) | 0.047** | 3.04 (1.01 -9.16) | 0.047** |
| Cidade de Maputo | 1.77 (0.63 – 4.96) | 0.273 | 1.70 (0.61 – 4.72) | 0.306 |

*Ref****+*** *includes respondent who had never experienced IPV and those who experienced IPV in the past 12 months.*
