# Supplementary material for: Intimate partner violence and common mental disorders among women in Mozambique: Evidence from the 2022-2023 Mozambique Demographic and Health Survey
Source: PLOS Ment Health. 2026 Oct 13;3(10):e0000660. doi: 10.1371/journal.pmen.0000660 (PMC13660890; doi:10.1371/journal.pmen.0000660)
Supplement: Supplementary file 2 [file pmen.0000660.s002.docx]

**S2 Table.** Weighted Percentage of Depression and Anxiety Comorbidity

|  | No anxiety | Anxiety Symptoms | Total |
| --- | --- | --- | --- |
| No depression | 2,534 (86.5%) | 84 (4.8%) | 2618 (91.4%) |
| Probable depression | 64 (2.8%) | **108 (5.8%)** | 172 (8.6%) |
| **Total** | 2598 (89.3%) | 192 (10.7%) | 2790 (100%) |
