# Supplementary material for: Intimate partner violence and common mental disorders among women in Mozambique: Evidence from the 2022-2023 Mozambique Demographic and Health Survey
Source: PLOS Ment Health. 2026 Oct 13;3(10):e0000660. doi: 10.1371/journal.pmen.0000660 (PMC13660890; doi:10.1371/journal.pmen.0000660)
Supplement: Supplementary file 3 [file pmen.0000660.s003.docx]

**S3 Table.** Multicollinearity Test of the Study Variables

| **Variable** | **Category** | **VIF** | **Tolerance (1/VIF)** |
| --- | --- | --- | --- |
| Physical violence | Yes | 1.32 | 0.76 |
| Sexual violence | Yes | 1.17 | 0.85 |
| Emotional violence | Yes | 1.32 | 0.76 |
| Age group (v013) | 20–24 | 2.55 | 0.39 |
|  | 25–29 | 2.66 | 0.38 |
|  | 30–34 | 2.41 | 0.41 |
|  | 35–39 | 2.40 | 0.42 |
|  | 40–44 | 2.09 | 0.48 |
|  | 45–49 | 1.84 | 0.54 |
| Region (v024) | Region 2 | 2.32 | 0.43 |
|  | Region 3 | 2.04 | 0.49 |
|  | Region 4 | 1.83 | 0.55 |
|  | Region 5 | 2.64 | 0.38 |
|  | Region 6 | 2.16 | 0.46 |
|  | Region 7 | 2.52 | 0.40 |
|  | Region 8 | 2.22 | 0.45 |
|  | Region 9 | 2.30 | 0.43 |
|  | Region 10 | 2.69 | 0.37 |
|  | Region 11 | 2.34 | 0.43 |
| Residence (v025) | Rural | 2.10 | 0.48 |
| Education (v106) | Primary | 1.67 | 0.60 |
|  | Secondary | 2.41 | 0.42 |
|  | Higher | 1.36 | 0.74 |
| Wealth index (v190) | Poorer | 1.85 | 0.54 |
|  | Middle | 2.13 | 0.47 |
|  | Richer | 3.00 | 0.33 |
|  | Richest | 5.16 | 0.19 |
| Marital status | Currently married | 1.33 | 0.75 |
| Religion | Category 2 | 1.79 | 0.56 |
|  | Category 6 | 1.11 | 0.90 |
| Media exposure | Yes | 1.54 | 0.65 |
| Respondent occupation | Working | 1.30 | 0.77 |
| Decision-making index | Medium | 1.25 | 0.80 |
|  | High | 1.29 | 0.78 |
| VIF Properties | Mean VIF | 2.06 |  |
|  | Minimum VIF | 1.11 |  |
|  | Maximum VIF | 5.16 |  |

VIF = Variance Inflation Factor. Values above 10 are typically indicative of serious multicollinearity. All variables had VIF values below this threshold, suggesting no evidence of problematic multicollinearity.
