# Supplementary material for: Intimate partner violence and common mental disorders among women in Mozambique: Evidence from the 2022-2023 Mozambique Demographic and Health Survey
Source: PLOS Ment Health. 2026 Oct 13;3(10):e0000660. doi: 10.1371/journal.pmen.0000660 (PMC13660890; doi:10.1371/journal.pmen.0000660)
Supplement: Supplementary file 4 [file pmen.0000660.s006.docx]

**Authors Response to Reviewer Comments**

Association between Intimate Partner Violence and Common Mental Disorders among Partnered Women in Mozambique: Results from the 2022-2023 Mozambique Demographic and Health Survey

Journal: PLOS Mental Health	Revision Date: 31st June, 2026

Authors: Bernard Afriyie Owusu, Benedict Weobong

| Comment | Response | Page # |
| --- | --- | --- |
| **Journal Specific Requirements**  For studies involving third-party data, we encourage authors to share any data specific to their analyses that they can legally distribute. PLOS recognizes, however, that authors may be using third-party data they do not have the rights to share. When third-party data cannot be publicly shared, authors must provide all information necessary for interested researchers to apply to gain access to the data.  For any third-party data that the authors cannot legally distribute, they should include the following information in their Data Availability Statement upon submission:  1) A description of the data set and the third-party source  2) If applicable, verification of permission to use the data set  3) Confirmation of whether the authors received any special privileges in accessing the data that other researchers would not have  4) All necessary contact information others would need to apply to gain access to the data | Thank you for this comment. We have revised our data availability statement to reflect the journal requirements.   **Revision** The data used in this study is from the Mozambique Demographic and Health Survey (MDHS). This is a nationally representative household survey conducted under the DHS Program. The data is owned and managed by the DHS Program and are available to researchers upon request. The authors obtained permission from the DHS Program to access and use the Mozambique DHS dataset for this study. The authors did not receive any special privileges or exclusive access to the data beyond the standard approval process available to all eligible researchers. Researchers who wish to access the MZDHS can register and submit a data access request through the DHS Program website. Information on registration and data access procedures is available from https://dhsprogram.com. For further inquiries, researchers may contact the DHS Program through the contact information provided on the official website. | 29 |
| ***Graphical Abstract:*** Please confirm whether you drew the images / clip-art within the figure panels by hand. If you did not draw the images, please provide (a) a link to the source of the images or icons and their license / terms of use; or (b) written permission from the copyright holder to publish the images or icons under our CC-BY 4.0 license. Alternatively, you may replace the images with open source alternatives. See these open source resources you may use to replace images / clip-art: | Thank you for this comment. The Authors confirm that they drew/developed the graphical abstract using Microsoft PowerPoint. | Graphical abstract |
| ***Graphical Abstract:*** please (a) provide a direct link to the base layer of the map (i.e., the country or region border shape) and ensure this is also included in the figure legend; and (b) provide a link to the terms of use / license information for the base layer image or shapefile. We cannot publish proprietary or copyrighted maps (e.g. Google Maps, Mapquest) and the terms of use for your map base layer must be compatible with our CC-BY 4.0 license.  Note: if you created the map in a software program like R or ArcGIS, please locate and indicate the source of the basemap shapefile onto which data has been plotted.  If your map was obtained from a copyrighted source please amend the figure so that the base map used is from an openly available source. Alternatively, please provide explicit written permission from the copyright holder granting you the right to publish the material under our CC-BY 4.0 license | Thank you for bringing this to our attention. The base map has been revised using Natural Earth Admin 0 – Countries (1:10m) dataset, and plotted in Stata 18/MP. The figure legend has been updated to include the source of the basemap, a direct link to the shapefile (or map source), and the applicable license information, which is compatible with the journal's CC BY 4.0 requirements.  Map of Africa highlighting Mozambique. Base map developed from Natural Earth (Public Domain) and Available at https://www.naturalearthdata .com/downloads/10m-cultural-vectors/10m-admin-0-countries/. and the Natural Earth  terms of use are available at https://www.naturalearthdata. com/about/terms-of-use/. | Graphical Abstract |
| 3. Please provide separate figure files in .tif or .eps format. | Thank you, we have provided separate figure files in tif. | All Figures |
| Please ensure all the standard sections present, (e.g. Abstract, Introduction, Results, Discussion, Methods), in the correct order, and that any references to subheadings in your main text are correct. An outline of the required sections can be consulted in our submission guidelines here: | All the standard sections are presented in the correct order | Manuscript |
| We notice that your supplementary tables are included in the manuscript file. Please remove them and upload them with the file type 'Supporting Information'. Please ensure that each Supporting Information file has a legend listed in the manuscript after the references list. | Thank you, this has been removed and uploaded as a supporting information.  We have also listed the supporting information after the reference list | 32 |
| **Reviewer 1**  The introduction is good and clear, but very extensive. I recommend trimming it and focusing on parts that are necessary to understand the results. | Thank you for this comment, we have revised the introductory section to make it concise. | 3-4 |
| PHQ-9 and GAD-7 are auxiliary diagnostic tools, but by themselves do not diagnose depression and anxiety. Thus, I suggest changing the language to depressive and anxiety symptoms. ` | We thank the reviewer for this helpful comment and agree that the screening instruments used in this study are not intended to establish a clinical diagnosis of depression or anxiety. For this reason, throughout the protocol we have deliberately referred to the outcomes as 'probable depression' and 'probable anxiety', terminology that is widely used and accepted in psychiatric epidemiology and global mental health research when validated screening scales are used to identify individuals likely to meet diagnostic criteria.  The measures employed in this study are screening scales that assess the underlying constructs of depression and anxiety by capturing a constellation of clinically relevant symptoms. While the term 'depressive and anxiety symptoms' is technically accurate, it does not fully reflect the purpose of these scales, which is to identify individuals at elevated likelihood of having the condition based on established cut-off scores rather than to quantify symptom severity alone. Referring to 'probable depression' and 'probable anxiety' therefore more accurately conveys the intended interpretation of the study outcomes while acknowledging that diagnostic confirmation would require a structured clinical assessment.  To avoid any ambiguity, we have revised the protocol to consistently state that the primary mental health outcomes are probable depression and probable anxiety, as identified using validated screening instruments, rather than clinically confirmed diagnoses. |  |
| The second paragraph of Potential confounders seems more like a result than a method. | Thank you for this observation. We developed the second paragraph to justify the choice of study variables. We have updated this by citing literature sources. | 9 |
| What are the educational categories? Is it years of study or primary, middle, high school-based? I ask because 74% attained a primary level, but 64% were in the middle to rich categories, and usually, more wealth equals more formal education. | The educational categories are: no education; primary; secondary; and higher. Per the results, 43% attained primary education, 23% attained secondary, and 2.7% attained higher education. In the narrative prior to the table, we stated that 74% had attained a *primary level or lower education* because primary plus no education = 74%.   Similar style was used to describe wealth. We note that majority of the respondents were in the middle to rich categories. This excludes the poorest and poorer categories. | 11 |
| The reporting could be more consistent; the percentages were presented as %, 3 out of 10, and written "setenvy percent." Table 1 could have a more in-depth legend. What is the unweighted, and what is the weighted? | We have revised the narrative to provide a consistent reporting in line with the proportions. That is, we currently report the percentages instead of the ratio.  We have provided the legend for the unweighted n and weighted % as below  *unweighted n: actual frequencies or sample size*  *weighted %: complex survey weighted proportions* | 11 |
| Figures 3 and 4 could be one figure. And Figure 5 is not necessary, since there's only one piece of information on it.  Table 3 could have a legend explaining the adjustment in the OR and what the ' Ref ' is on the values. | Yes, we have revised Figures 3 and 4 into one figure and replaced Figure 5 with a new Figure which provides a more comprehensive information regarding the prevalence of anxiety symptoms, depressive symptoms, comorbidity, and CMD.  We have spelt out the OR, and the adjustments are the list of variables provided. We have also provided a legend to explain the ‘Ref’ (Reference category) | 20 |
| Correct the causal language, focus on the association of IPV and CMDs; from the statistical methods, the authors can not infer causality. Likewise, when discussing the economic resources, the authors write that wealth protected women from CMDs, but they did not test it. | These have been corrected. Thank you. | 23 |
| Since the regions differed so much, I suggest that the authors test the CMD by regions, which can corroborate the hypothesis that more IPV, more CMD. How it is written seems to suggest that the regions themselves are causing higher CMD.  Similarly, the authors can correct the statistical tests using the women not cohabitating to see if the CMD levels are different, which can strengthen the IPV results. | We thank the reviewer for this suggestion. We examined the regional distribution of both IPV and poor mental health as suggested (results not presented). Our analysis showed that, the spatial patterns were not identical across regions. Some regions with high IPV prevalence did not exhibit correspondingly high levels of poor mental health. We have revised the language in the discussion section  The IPV model was only administered to women in union, and this precludes never married women. As such, we are unable to test to compare CMD by union and non-union types. | 24 |
| Conclusion: Again, limit the causal language. | Causal language revised | 28 |
| **Reviewer 2**  Did the authors propose any explanation for the finding that joint household decision-making was associated with increased odds of common mental disorders (CMDs)? | Yes, in the 5th paragraph, we emphasised on shifting gender norms, shared decision-making conflict, reduced autonomy, and patriarchy. Again, in line with the suggestions from the third reviewer, we have also provided alternative explanations regarding the mental load of shared decision making, selective effects, and economic reasons. | 24 |
| The Introduction would benefit from greater conciseness. The discussion of the Ecological Model of Violence (EMV) and Intersectionality Theory appears more relevant to the interpretation of the results and could therefore be moved to the Discussion section. | Thank you for this comment, we have revised the introductory section and moved some of the discussion on the theories to the discussion section as suggested. | 23, 25 |
| Could the authors provide additional information regarding the Mozambique Demographic and Health Survey (MDHS), including the period during which the survey was conducted?  Furthermore, in the "Data and Sampling" section, the authors should clearly report the number of participants initially assessed, as well as the numbers included and excluded from the final analysis, together with the reasons for exclusion. | We have provided more information about the period of the MZDHS including “… The MDHS is implemented by the Mozambique National Institute of Statistics (INE), in collaboration with the Health Ministry with technical support from the DHS Programme. Field work for the MDHS 2022 – 2023 was conducted from 27th July, 2022 to 27th February, 2023 [20]”.   Thank you for this suggestion. Our Figure 2 is intended to address this concern. | 6  Fig 2 |
| The authors should consider adding a brief overview of Major Depressive Disorder (MDD) and Anxiety Disorders in the Introduction, with particular emphasis on epidemiological data from Africa. This would provide important context regarding the burden of common mental disorders in the region and further justify the study. | We have added a brief overview of MADD as stated below …which is a comorbid presentation of anxiety and depression… In sub-Saharan Africa, these conditions are pronounced due to intersecting social, economic, and structural vulnerabilities; however, anxiety and depression are often conceptualised as distinct diagnostic conditions despite their frequent co-occurrence in population-based studies. | 5 |
| Did the authors consider collecting primary data through questionnaire administration rather than relying exclusively on secondary data from the MDHS? | Thank you for this comment. No, we analysed the secondary data, and did not collect primary data. However, we have made recommendations for further research using mixed methods. | 27 |
| **Reviewer 3**  The title is unnecessarily long. The authors should consider a more concise title that highlights the main objective and population. | We thank the reviewer for this comment. We have provided a concise title which is  “**Intimate Partner Violence and Common Mental Disorders among Women in Mozambique: Evidence from the 2023 Demographic and Health Survey**” | 1 |
| The authors combined depression and anxiety into a single CMD outcome. Please provide a stronger justification for this approach and discuss its implications for interpreting the results. | Thank you so much for this observation as it forms a key part of the gap identified. We have provided some justifications in the last part of the introduction which reads “.. we note that IPV survivors typically experience mixed anxiety-depressive disorders (MADD), which is a comorbid presentation of anxiety and depression, with evidence demonstrating that sexual, physical, and psychological IPV’s produce mental health effects that are better captured through integrated assessment [12,17]. In SSA, MADD is pronounced due to intersecting social, economic, and structural vulnerabilities. Yet, anxiety and depression are often conceptualised as distinct diagnostic conditions despite their frequent co-occurrence in population-based studies.”  Based on this, we have provided an implication for interpreting the results under the strengths and limitations section “Combining depression and anxiety into a single CMD outcome implies that the results reflect associations with general psychological distress (common mental disorders) rather than disorder-specific effects.” | 5,7  26 |
| The Discussion attributes stronger effects to recent IPV than past IPV. Given the cross-sectional design, the authors should interpret these findings with greater caution and avoid making temporal inferences. | This is well noted. We have acknowledged this in the limitation section “The observed stronger association between IPV and CMDs compared with past IPV may reflect differential reporting or recall patterns rather than a clear temporal gradient, and should therefore be interpreted with caution” | 27 |
| The finding that joint household decision-making increases the odds of CMDs is unexpected. The authors should explore alternative explanations and discuss this result in greater depth. | Thank you for this comment. Beyond patriarchy, and conflict perspectives, we have added decision-making burden, cognitive and emotional workload and selection effects as alternative explanations. | 23 |
| The manuscript frequently refers to intersectionality. However, marital status was the only significant interaction term. The authors should better align the discussion with the statistical findings. | We thank the reviewer for this comment. Yes, although all the variables were positively associated with CMD as seen in Fig 6, only marital status was statistically significant at the 0.05 threshold. We have therefore revised the language to   “While intersectionality theory offers a useful framework for understanding how multiple social positions interact to influence CMD outcomes, the study provides partial empirical support for intersectionality by demonstrating that certain socio-economic positions, particularly marital contexts” | 25 & abstract |
| Emotional IPV showed the strongest association with CMDs. The Discussion should more clearly explain the public health implications of this finding and how it may inform screening and prevention strategies. | Thank you for this comment. The 3rd paragraph of the discussion is focused on emotional IPV. We also noted this important concern at the section on policy and implications | 22,26 |

We are so much grateful to the Reviewers for their review and timely feedback.

Bernard and Benedict
