# Supplementary material for: Intimate partner violence and common mental disorders among women in Mozambique: Evidence from the 2022-2023 Mozambique Demographic and Health Survey
Source: PLOS Ment Health. 2026 Oct 13;3(10):e0000660. doi: 10.1371/journal.pmen.0000660 (PMC13660890; doi:10.1371/journal.pmen.0000660)
Supplement: Peer review history

# Peer review: Decision Letter 0

**DOI:** 10.1371/journal.pmen.0000660.r001

**Authors:** Aline Siteneski

29 Jun 2026

PMEN-D-26-00311

Association between Intimate Partner Violence and Common Mental Disorders among Partnered Women in Mozambique: Results from the 2022-2023 Mozambique Demographic and Health Survey

PLOS Mental Health

Dear Dr. Owusu,

Thank you for submitting your manuscript to PLOS Mental Health. After careful consideration, we feel that it has merit but does not fully meet PLOS Mental Health’s publication criteria as it currently stands. Therefore, we invite you to submit a revised version of the manuscript that addresses the points raised during the review process.

Please submit your revised manuscript by Aug 28 2026 11:59PM. If you will need more time than this to complete your revisions, please reply to this message or contact the journal office at mentalhealth@plos.org. When you're ready to submit your revision, log on to https://www.editorialmanager.com/pmen/ and select the 'Submissions Needing Revision' folder to locate your manuscript file.

Please include the following items when submitting your revised manuscript:

- A letter that responds to each point raised by the editor and reviewer(s). You should upload this letter as a separate file labeled 'Response to Reviewers'.
- A marked-up copy of your manuscript that highlights changes made to the original version. You should upload this as a separate file labeled 'Revised Manuscript with Track Changes'.
- An unmarked version of your revised paper without tracked changes. You should upload this as a separate file labeled 'Manuscript'.

Guidelines for resubmitting your figure files are available below the reviewer comments at the end of this letter.

As the corresponding author, your ORCID iD is verified in the submission system and will appear in the published article. PLOS supports the use of ORCID, and we encourage all coauthors to register for an ORCID iD and use it as well. Please encourage your coauthors to verify their ORCID iD within the submission system before final acceptance, as unverified ORCID iDs will not appear in the published article. *Only* the individual author can complete the verification step; PLOS staff *cannot* verify ORCID iDs on behalf of authors.

We look forward to receiving your revised manuscript.

Kind regards,

Aline Siteneski, Ph.D.

Academic Editor

PLOS Mental Health

**Journal Requirements:**

1. For studies involving third-party data, we encourage authors to share any data specific to their analyses that they can legally distribute. PLOS recognizes, however, that authors may be using third-party data they do not have the rights to share. When third-party data cannot be publicly shared, authors must provide all information necessary for interested researchers to apply to gain access to the data. (https://journals.plos.org/plosone/s/data-availability#loc-acceptable-data-access-restrictions)

For any third-party data that the authors cannot legally distribute, they should include the following information in their Data Availability Statement upon submission:

1) A description of the data set and the third-party source

2) If applicable, verification of permission to use the data set

3) Confirmation of whether the authors received any special privileges in accessing the data that other researchers would not have

4) All necessary contact information others would need to apply to gain access to the data

2. Some material included in your submission may be copyrighted. According to PLOS’s copyright policy, authors who use figures or other material (e.g., graphics, clipart, maps) from another author or copyright holder must demonstrate or obtain permission to publish this material under the Creative Commons Attribution 4.0 International (CC BY 4.0) License used by PLOS journals. Please closely review the details of PLOS’s copyright requirements here: PLOS Licenses and Copyright. If you need to request permissions from a copyright holder, you may use PLOS's Copyright Content Permission form.

Please respond directly to this email or email the journal office and provide any known details concerning your material's license terms and permissions required for reuse, even if you have not yet obtained copyright permissions or are unsure of your material's copyright compatibility.

Potential Copyright Issues:

a. Graphical Abstract: Please confirm whether you drew the images / clip-art within the figure panels by hand. If you did not draw the images, please provide (a) a link to the source of the images or icons and their license / terms of use; or (b) written permission from the copyright holder to publish the images or icons under our CC-BY 4.0 license. Alternatively, you may replace the images with open source alternatives. See these open source resources you may use to replace images / clip-art:

- https://commons.wikimedia.org

- https://openclipart.org/

b. Graphical Abstract: please (a) provide a direct link to the base layer of the map (i.e., the country or region border shape) and ensure this is also included in the figure legend; and (b) provide a link to the terms of use / license information for the base layer image or shapefile. We cannot publish proprietary or copyrighted maps (e.g. Google Maps, Mapquest) and the terms of use for your map base layer must be compatible with our CC-BY 4.0 license.

Note: if you created the map in a software program like R or ArcGIS, please locate and indicate the source of the basemap shapefile onto which data has been plotted.

If your map was obtained from a copyrighted source please amend the figure so that the base map used is from an openly available source. Alternatively, please provide explicit written permission from the copyright holder granting you the right to publish the material under our CC-BY 4.0 license.

Please note that the following CC BY licenses are compatible with PLOS license: CC BY 4.0, CC BY 2.0 and CC BY 3.0, meanwhile such licenses as CC BY-ND 3.0 and others are not compatible due to additional restrictions.

If you are unsure whether you can use a map or not, please do reach out and we will be able to help you. The following websites are good examples of where you can source open access or public domain maps:

* U.S. Geological Survey (USGS) - All maps are in the public domain. (http://www.usgs.gov)

* PlaniGlobe - All maps are published under a Creative Commons license so please cite “PlaniGlobe, http://www.planiglobe.com, CC BY 2.0” in the image credit after the caption. (http://www.planiglobe.com/?lang=enl)

* Natural Earth - All maps are public domain. (http://www.naturalearthdata.com/about/terms-of-use/)

3. Please provide separate figure files in .tif or .eps format.

For more information about figure files please see our guidelines:

https://journals.plos.org/mentalhealth/s/figures

https://journals.plos.org/mentalhealth/s/figures#loc-file-requirements

4. Please ensure all the standard sections present, (e.g. Abstract, Introduction, Results, Discussion, Methods), in the correct order, and that any references to subheadings in your main text are correct. An outline of the required sections can be consulted in our submission guidelines here:

https://journals.plos.org/mentalhealth/s/submission-guidelines#loc-parts-of-a-submission

5.  We notice that your supplementary tables are included in the manuscript file. Please remove them and upload them with the file type 'Supporting Information'. Please ensure that each Supporting Information file has a legend listed in the manuscript after the references list.

If the reviewer comments include a recommendation to cite specific previously published works, please review and evaluate these publications to determine whether they are relevant and should be cited. There is no requirement to cite these works unless the editor has indicated otherwise.

**Additional Editor Comments (if provided):**

[Note: HTML markup is below. Please do not edit.]

Reviewers' comments:

Reviewer's Responses to Questions

**Comments to the Author**

1. Does this manuscript meet PLOS Mental Health’s publication criteria?

Reviewer #1: Yes

Reviewer #2: Yes

Reviewer #3: Yes

**********

2. Has the statistical analysis been performed appropriately and rigorously?

Reviewer #1: Yes

Reviewer #2: Yes

Reviewer #3: Yes

**********

3. Have the authors made all data underlying the findings in their manuscript fully available (please refer to the Data Availability Statement at the start of the manuscript PDF file)?

The PLOS Data policy

Reviewer #1: Yes

Reviewer #2: Yes

Reviewer #3: Yes

**********

4. Is the manuscript presented in an intelligible fashion and written in standard English?

Reviewer #1: Yes

Reviewer #2: Yes

Reviewer #3: Yes

**********

**Reviewer #1:**  The study delves into how IPV is associated with CMDs using national data from Mozambique. The authors use an ecological model of violence and intersectionality theory to understand how different women suffering emotional, physical, or sexual IPV show CMD. It is a very important and interesting topic, and the work is appropriate, but I have some concerns that need to be clarified and addressed to improve the study.

Introduction

The introduction is good and clear, but very extensive. I recommend trimming it and focusing on parts that are necessary to understand the results.

PHQ-9 and GAD-7 are auxiliary diagnostic tools, but by themselves do not diagnose depression and anxiety. Thus, I suggest changing the language to depressive and anxiety symptoms.

Methods

The second paragraph of Potential confounders seems more like a result than a method.

Results

What are the educational categories? Is it years of study or primary, middle, high school-based? I ask because 74% attained a primary level, but 64% were in the middle to rich categories, and usually, more wealth equals more formal education.

The reporting could be more consistent; the percentages were presented as %, 3 out of 10, and written "setenvy percent."

Table 1 could have a more in-depth legend. What is the unweighted, and what is the weighted?

Figures 3 and 4 could be one figure. And Figure 5 is not necessary, since there's only one piece of information on it.

Table 3 could have a legend explaining the adjustment in the OR and what the ' Ref ' is on the values.

Discussion

Correct the causal language, focus on the association of IPV and CMDs; from the statistical methods, the authors can not infer causality. Likewise, when discussing the economic resources, the authors write that wealth protected women from CMDs, but they did not test it.

Since the regions differed so much, I suggest that the authors test the CMD by regions, which can corroborate the hypothesis that more IPV, more CMD. How it is written seems to suggest that the regions themselves are causing higher CMD.

Similarly, the authors can correct the statistical tests using the women not cohabitating to see if the CMD levels are different, which can strengthen the IPV results.

Conclusion

Again, limit the causal language.

**Reviewer #2:**  • Did the authors propose any explanation for the finding that joint household decision-making was associated with increased odds of common mental disorders (CMDs)?

• The Introduction would benefit from greater conciseness. The discussion of the Ecological Model of Violence (EMV) and Intersectionality Theory appears more relevant to the interpretation of the results and could therefore be moved to the Discussion section.

• Could the authors provide additional information regarding the Mozambique Demographic and Health Survey (MDHS), including the period during which the survey was conducted? Furthermore, in the "Data and Sampling" section, the authors should clearly report the number of participants initially assessed, as well as the numbers included and excluded from the final analysis, together with the reasons for exclusion.

• The authors should consider adding a brief overview of Major Depressive Disorder (MDD) and Anxiety Disorders in the Introduction, with particular emphasis on epidemiological data from Africa. This would provide important context regarding the burden of common mental disorders in the region and further justify the study.

• Did the authors consider collecting primary data through questionnaire administration rather than relying exclusively on secondary data from the MDHS?

**Reviewer #3:**  The manuscript entitled Association between Intimate Partner Violence and Common Mental Disorders among Partnered Women in Mozambique: Results from the 2022–2023 Mozambique Demographic and Health Survey addresses an important public health issue and provides valuable national evidence from Mozambique. The study is relevant and well-positioned to inform policy and practice. However, several aspects require clarification to strengthen the interpretation of the findings.

Comments:

1. The title is unnecessarily long. The authors should consider a more concise title that highlights the main objective and population.

2. The authors combined depression and anxiety into a single CMD outcome. Please provide a stronger justification for this approach and discuss its implications for interpreting the results.

3. The Discussion attributes stronger effects to recent IPV than past IPV. Given the cross-sectional design, the authors should interpret these findings with greater caution and avoid making temporal inferences.

4. The finding that joint household decision-making increases the odds of CMDs is unexpected. The authors should explore alternative explanations and discuss this result in greater depth.

5. The manuscript frequently refers to intersectionality. However, marital status was the only significant interaction term. The authors should better align the discussion with the statistical findings.

6. Emotional IPV showed the strongest association with CMDs. The Discussion should more clearly explain the public health implications of this finding and how it may inform screening and prevention strategies.

**********

what does this mean?). If published, this will include your full peer review and any attached files.

**Do you want your identity to be public for this peer review?** If you choose “no”, your identity will remain anonymous but your review may still be made public.

For information about this choice, including consent withdrawal, please see our Privacy Policy

Reviewer #1: No

Reviewer #2: No

Reviewer #3: **Yes:**  Fabio Serra Barbosa da Silva

**********

[NOTE: If reviewer comments were submitted as an attachment file, they will be attached to this email and accessible via the submission site. Please log into your account, locate the manuscript record, and check for the action link "View Attachments". If this link does not appear, there are no attachment files.]

**Figure Resubmissions:**

# Author comment: Author response to Decision Letter 1

**DOI:** 10.1371/journal.pmen.0000660.r002

1 Jul 2026

Attachment Submitted filename: Response to Reviewers bao_bw_bao.docx

# Peer review: Decision Letter 1

**DOI:** 10.1371/journal.pmen.0000660.r003

**Authors:** Aline Siteneski

19 Aug 2026

Intimate Partner Violence and Common Mental Disorders among Women in Mozambique: Evidence from the 2022-2023 Mozambique Demographic and Health Survey

PMEN-D-26-00311R1

Dear Mr. Owusu,

We are pleased to inform you that your manuscript 'Intimate Partner Violence and Common Mental Disorders among Women in Mozambique: Evidence from the 2022-2023 Mozambique Demographic and Health Survey' has been provisionally accepted for publication in PLOS Mental Health.

Before your manuscript can be formally accepted you will need to complete some formatting changes, which you will receive in a follow up email. A member of our team will be in touch with a set of requests.

Please note that your manuscript will not be scheduled for publication until you have made the required changes, so a swift response is appreciated.

IMPORTANT: The editorial review process is now complete. PLOS will only permit corrections to spelling, formatting or significant scientific errors from this point onwards. Requests for major changes, or any which affect the scientific understanding of your work, will cause delays to the publication date of your manuscript.

If your institution or institutions have a press office, please notify them about your upcoming paper to help maximize its impact. If they'll be preparing press materials, please inform our press team as soon as possible -- no later than 48 hours after receiving the formal acceptance. Your manuscript will remain under strict press embargo until 2 pm Eastern Time on the date of publication. For more information, please contact mentalhealth@plos.org.

Thank you again for supporting Open Access publishing; we are looking forward to publishing your work in PLOS Mental Health.

Best regards,

Aline Siteneski, Ph.D.

Academic Editor

PLOS Mental Health

***********************************************************

The revised manuscript entitled “Intimate Partner Violence and Common Mental Disorders among Women in Mozambique: Evidence from the 2022–2023 Mozambique Demographic and Health Survey” (Manuscript ID: PMEN-D-26-00311R1) has been carefully evaluated following revision. The authors have satisfactorily addressed the main concerns raised during peer review. Only a few minor changes suggested by the reviewers remain, which should be incorporated in the final version. I am pleased to accept the manuscript for publication, subject to these minor editorial corrections.

Reviewer Comments (if any, and for reference):

Reviewer's Responses to Questions

**Comments to the Author**

Reviewer #1: All comments have been addressed

Reviewer #3: All comments have been addressed

**********

publication criteria?

Reviewer #1: Yes

Reviewer #3: Yes

**********

3. Has the statistical analysis been performed appropriately and rigorously?

Reviewer #1: Yes

Reviewer #3: Yes

**********

4. Have the authors made all data underlying the findings in their manuscript fully available (please refer to the Data Availability Statement at the start of the manuscript PDF file)?

The PLOS Data policy

Reviewer #1: Yes

Reviewer #3: Yes

**********

5. Is the manuscript presented in an intelligible fashion and written in standard English?

Reviewer #1: Yes

Reviewer #3: Yes

**********

Reviewer #1: I thank the authors for their responses to the other reviewers and me; the text has greatly improved.

There are minor typing errors; I recommend a review of the text.

Reviewer #3: 1. Please revise the remaining causal terminology. Expressions such as “protective,” “impact,” or “increases risk” should be replaced with association-based language consistent with the cross-sectional design.

2. Please use “probable depression” and “probable anxiety” consistently because PHQ-9 and GAD-7 are screening instruments rather than diagnostic assessments.

3. The Discussion still suggests that the weaker association with past IPV may reflect attenuation, resilience, or recovery over time. These temporal explanations should remain explicitly speculative.

4. The interpretation of intersectionality should consistently emphasize partial empirical support, because marital status was the only socio-economic characteristic showing significant effect modification.

5. Please clarify that the adjusted estimates for recent IPV derive from different regression models. The aOR of 2.05 comes from the three-category IPV model, whereas 2.29 comes from the binary recent-IPV model.

**********

what does this mean?). If published, this will include your full peer review and any attached files.

**Do you want your identity to be public for this peer review?** If you choose “no”, your identity will remain anonymous but your review may still be made public.

For information about this choice, including consent withdrawal, please see our Privacy Policy

Reviewer #1: No

Reviewer #3: **Yes:**  Fabio Serra Barbosa da Silva

**********
